# Neuroplastic Mechanisms Underlying Perceptual and Cognitive Enhancement

**DOI:** 10.1155/2016/6238571

**Published:** 2016-05-15

**Authors:** Etienne de Villers-Sidani, Jyoti Mishra, Xiaoming Zhou, Patrice Voss

**Affiliations:** ^1^McGill University, Montreal, QC, Canada H3A 0G4; ^2^University of California, San Francisco, CA, USA; ^3^East China Normal University, Shanghai 200062, China

The remarkable plastic nature of our brain, often manifesting itself via changes in how brain circuits code information, is at the origin of the many complex skills we master both during early development and adulthood. Intensive work in the last few decades in both human and animal models has revealed the multiple facets of brain plasticity and culminated recently in the explosion of the field of cognitive neurotherapeutics, bringing hope that brain plasticity can be used for the remediation of a wide range of cognitive, perceptual, or motor deficits.

This growing interest in brain plasticity now shared by the general public has however shed light on the fact that our understanding of the regulating mechanisms of plasticity in the young, adult, and aging brain and how it can be best harnessed for therapeutic purposes remains poor. This limitation stands as a significant roadblock in the elaboration of effective science-based strategies for the remediation of neurological impairments at all ages and the preservation of optimal brain function in older adults.

The objective of this special issue is to bring further attention to the field of neuroplasticity and cognitive neurotherapeutics by presenting novel original work performed in humans and animal models focusing on the impact of experience on brain circuits and behavior.

Studying plasticity in sensory and motor systems is a particularly powerful means of understanding how brain circuits are shaped by experience. This is reflected in this issue which features several articles related to adaptive and maladaptive sensory or motor learning in various clinical populations. For example, M. M. Shiell et al. demonstrate that structural plasticity in a typical auditory cortical region, the right planum temporale, supports enhanced visual performance (motion detection) in deaf individuals, the first evidence of a neuroanatomical marker of crossmodal plasticity in this population. In the same vein, M. S. Houde et al. present an overview on the effects of deafness on body-related (nonvisual) processes, by reinterpreting the current literature on the altered processing as result of deafness from the “body” point of view. In their article, I. Riquelme et al. studied somatosensory processing in children with autism spectrum disorder. They evidenced using standardized tests that these individuals have predominantly abnormal processing of painful stimuli, implying a selective dysfunction of type C unmyelinated sensory fibers. These findings could provide a novel basis for the elaboration of sensory remediation strategies in this clinical population. P. Voss et al. examined the role of cholinergic system augmentation on auditory learning in young and older rats. Their results show that the speed and specificity of learning is significantly enhanced by the administration of rivastigmine in both young and older animals. Their findings also demonstrate that the rules of auditory cortical plasticity could be substantially different in the aging brain. Concerned with the mechanisms underlying auditory brainstem plasticity, H.-X. Mei et al. demonstrated that activity in the commissure of the inferior colliculus has a strong influence on the tuning properties of inferior collicular neurons. Their results could have important implications for the processing and integration of binaural interactions.

The ability to shape “higher order” brain processes such as cognitive control with training is also now well recognized. In this issue, A. J. Wilkinson and L. Yang explore learning and benefits of inhibition tasks in older adults and find improvements with practice, though transfer to other cognitive abilities was limited to other tasks that shared a similar task structure. In this study, training was limited to 2 weeks and so the effects could potentially be enhanced through increased training dose or by pairing with neurostimulation. S. R. Schroeder et al. demonstrated in a study involving more than 200 young healthy participants that bilingualism and musicianship improve executive control and could therefore potentially be used in rehabilitations programs to boost cognitive performance. H. Takeuchi et al. show the utility of training on fast simple numerical calculations; one week of training enhanced processing speed and executive function in young adults. These effects were mediated by structural plasticity and perfusion related changes in frontopolar cortex.

The development of noninvasive and safe techniques to augment brain plasticity such as transcranial direct current stimulation (tDCS) is very appealing for obvious reasons but their mechanisms of action and how to best use them are still not well established. Three studies presented in this special issue explored these questions. S. Sikström et al. examined whether tDCS would be more beneficial to individuals with low attentiveness compared to attentive people. Their results confirmed their hypotheses and strongly suggest that the benefits of tDCS likely interact with attentiveness. In another tDCS study, H. Kumru et al. investigated the use of mirror visual feedback (MVF) therapy as a tool to promote plastic brain changes and motor recovery. Their results show that a combination of motor training with MVF therapy may induce more robust neuroplastic changes through multisensory integration which is key for motor rehabilitation. Finally, G. Dumel et al. show that tDCS can significantly enhance motor learning in older adults raising the possibility that this technique could be useful to alleviate age-associated motor function decline.

The enhancement of basic sensory processing or complex cognitive function requires the participation of a large number of complex molecules, many of which are still unknown or have poorly understood function. Hypothalamic neuropeptides are among those brain compounds that currently receive attention because of their powerful ability to shape neural circuits and behavior in both the developing and adult brains. J. Bakos et al. explored in this issue the role of several hypothalamic neuropeptides including oxytocin in the regulation of adult neurogenesis and neuritogenesis. This review highlights the potential role of these small molecules in a number of brain circuits linked to key social behaviors. In another article focusing on the molecular foundation of learning B. Gómez-Chacón et al. examined the impact of N-ethylmaleimide-sensitive factor expression on plasticity in the amygdala and perirhinal cortex. Their data provide new insight into some of the fundamental mechanisms involved in building taste recognition memory through sensory experience. M. Kruk-Slomka et al. demonstrated in mice that the endocannabinoid system played a significant role in the regulation of oxidative stress and long-term memory formation. Finally, in a thoughtful review article, K. M. Bieszczad and M. L. Phan explore the role of epigenetic mechanisms in the formation and regulation of memory encoding in sensory cortices.

In summary, the articles contained in this issue highlight original findings obtained using a wide range of complementary techniques (fMRI, tDCS, single cell recordings, and pharmacology) in multiple brain systems and clinical populations that we believe will stimulate the continuing effort to better understand the relationship between brain plasticity, its regulators, and its impact on behavioral function.

*Etienne de Villers-Sidani* *Etienne de Villers-Sidani* *Jyoti Mishra* *Jyoti Mishra* *Xiaoming Zhou* *Xiaoming Zhou* *Patrice Voss* *Patrice Voss*

